# Ownership and use of insecticide-treated nets during pregnancy in sub-Saharan Africa: a review

**DOI:** 10.1186/1475-2875-12-268

**Published:** 2013-08-01

**Authors:** Megha Singh, Graham Brown, Stephen J Rogerson

**Affiliations:** 1 Nossal Institute for Global Health, University of Melbourne, Carlton, VIC, Australia; 2 Department of Medicine, University of Melbourne, Carlton, VIC, Australia

**Keywords:** Malaria prevention, Pregnancy, Insecticide-treated nets, *Plasmodium falciparum*

## Abstract

Over the past decade, significant gains have been made in the implementation of malaria prevention measures in pregnancy in sub-Saharan Africa, including the distribution of insecticide-treated nets (ITNs). These have been shown to cause a reduction in the incidence of malaria and its consequences such as maternal anaemia, stillbirths and intrauterine growth restriction. Currently most nations in Africa have policies for distributing ITNs to pregnant women through various mechanisms, however coverage remains well below the targets. This review summarizes recent evidence regarding the correlation between ownership and use of ITNs and the determinants of both, in pregnancy in sub-Saharan Africa, and reviews interventions directed at improving coverage. A review of the literature using Pubmed, CINAHL and scanning of reference lists was conducted in October 2012 and 59 articles were selected for final review. The research obtained was a mixture of national and district level surveys, and a narrative synthesis of the data was undertaken. Ownership of ITNs varied from as low as 3% to greater than 80%, and the main determinants were found to be education level, knowledge of malaria, community involvement, socio-economic status and parity, although the significance of each varied between the different settings and studies reviewed. In more than half the settings where data were available, the combination of lack of availability and lack of use of an available net meant that less than half of all pregnancies received the recommended intervention. Supply and cost remain major barriers to achieving optimal coverage, but the additional important contributor to reduced efficiency of intervention was the clear discrepancy between ownership and use, with available ITN use below 60% in several settings. Cited reasons for not using an ITN, where one was available, included discomfort, problems with hanging up nets and lack of space, low awareness of need, and seasonal variations in use. These findings highlight the need for context-specific approaches and educational components to be incorporated into ITN distribution programmes to address some of the reasons why some pregnant women do not use the ITNs they own.

## Background

The World Health Organization (WHO) 2011 World Malaria Report demonstrates the enormity of the burden of malaria, with 216 million cases and 655,000 deaths attributable to this mosquito-transmitted parasite in 2010 alone. The burden is largely borne by Africa where 91% of deaths occurred, with pregnant women and children under five years of age most at risk of infection and adverse outcomes [1]. Each year, there are an estimated 25 million pregnancies in sub-Saharan Africa at risk of malaria, the consequences of which can be serious for both mother and foetus in terms of morbidity and mortality. Sequelae of malaria in pregnancy include maternal anaemia, stillbirths, low birth weight and intrauterine growth restriction [2].

The main malaria prevention strategies in pregnancy include the use of intermittent preventative treatment with anti-malarial medications, as well as the regular and timely use of long-lasting, insecticide-treated nets (LLINs). The WHO and Roll Back Malaria (RBM) partnership now recommend that distribution of LLINs be free or heavily subsidized to achieve greater equity of coverage, and that a variety of distribution systems be used to achieve universal access, including targeted campaigns to deliver nets to most-at-risk populations, which include pregnant women and children under five years of age [3]. The investment into this simple yet effective intervention has been substantial. In 2010, there were enough insecticide-treated nets (ITNs), primarily LLINs, procured on the African continent to cover 73% of the at-risk population, yet achieving equitable distribution and providing ongoing supply remain a challenge [1,4].

The evidence for the efficacy of ITNs in preventing malaria infection and its consequences in pregnancy is strong, as reported in a Cochrane review in 2009 [5], and in a more recent meta-analysis [6], which examined malaria prevention in pregnancy datasets from different African nations. The evidence showed a strong correlation between the use of ITNs and reduction in stillbirths, improvements in birth weights of babies and a reduction in the prevalence of parasitaemia and anaemia in pregnant women. A communal protective effect of ITNs and reduction in overall vector density has also been observed in some settings [7,8].

Despite the widespread roll out of policies, and substantial financial investments in ITN distribution, coverage remains suboptimal in many regions, particularly with respect to pregnant women. Recently published data from sub-Saharan Africa found that although 96% of countries surveyed had a policy for ITN coverage, reported coverage of pregnant women with ITNs was only 17% [9]. The main delivery system for ITNs was through antenatal clinics (ANCs), using free distribution or a voucher system. Interestingly, attendance at an ANC was not found to be a major factor in limiting coverage. Supply has been identified by the WHO as the primary barrier to achieving optimal coverage, with the latest World Malaria Report suggesting that in the general population, there is a high correlation between ownership and use of ITNs (range 93-99%) [1]. This is despite wide variation in this relationship at a national level in the African region, as shown by Eisele *et al.* in their summary of demographic and health surveys (DHS) and malaria indicator surveys (MIS) from 15 countries [10].

The relationship between ITN ownership and use has been explored by only a few authors, and largely neglected when concerning pregnant women. A 2011 qualitative systematic review of the uptake of malaria prevention interventions in pregnancy in Africa found that relationships with health workers, cost and distance to health facilities, knowledge of antenatal care and local contextual factors influenced women’s uptake of interventions [11]. Pullford *et al.* also conducted a review regarding the reported reasons for not using a bed net when one was available (in the general population) and found that reasons such as discomfort and perceived low mosquito density were the most common reasons for non-use [12].

Given that pregnant women and their unborn children are a particularly vulnerable group susceptible to malaria and its consequences, a review of the current literature was conducted, to better clarify the relationship between ownership and use of ITNs, and the determinants of both, in this target population.

### Objectives

This review summarizes the body of evidence published in the last five years, regarding the determinants of ITN ownership and use in pregnant women in sub-Saharan Africa, and examines the relationship between these two outcomes. Successful strategies that have been employed to increase ITN coverage amongst pregnant women are identified, and key gaps in ownership and use that should be addressed are highlighted. In the past few years there has been an increased focus on, and improvements in, coverage in some areas, and thus a timely review, limited to the last five years, seemed to be most relevant for addressing the current coverage gaps.

### Definitions

In this review paper, the term ITN will refer to nets that have been treated with insecticide and need ongoing treatment, or LLINs, which are the most frequently distributed type of net in Africa [1].

Ownership is based on reported household ownership of an ITN, as per the DHS definition [13]. The RBM indicator for coverage is ‘the proportion of pregnant women who have slept under an ITN the previous night’ [14], and in this paper coverage has been used interchangeably with overall use. There remains an unclear definition of coverage in many studies, with some studies using ownership as a proxy for coverage (assuming all nets owned are used), and others using coverage as per the RBM definition. These definitions are distinct from ‘available ITN use’ (a key indicator in this review), which is expressed as the proportion of pregnant women who have slept under an ITN the previous night, when at least one ITN was available in the household. Universal coverage is achieved when all members of a household are protected by an ITN the previous night, at the optimal intrahousehold target of one ITN for every two members [3].

## Methods

A comprehensive search of literature was carried out using electronic databases Pubmed and CINAHL (2007–2012), as well as scanning reference lists in October 2012. The searches were limited to the English language, and to studies published from 2007 onwards. The CINAHL search excluded Pubmed articles. The search terms used were ITN; Bed Net; Mosquito Net and these were all combined using the boolean operator ‘AND’ with either pregnan*; strateg*; ownership; utilization; prevention; control; supply; and distribution (some were Medical Subject Headings).

**Figure 1:**
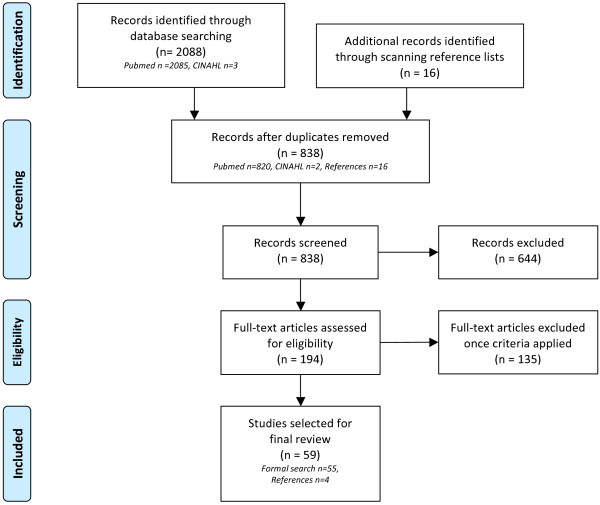
**Search strategy.** Adapted from the PRISMA 2009 Flow Chart [15].

The overall search strategy generated 838 articles, after duplicates were removed using the Endnote® data management programme. There were 820 articles from Pubmed, two from CINAHL, with a further 16 obtained from scanning reference lists. These 838 articles were screened by reading abstracts, and after applying inclusion and exclusion criteria (listed below), there were 194 articles remaining. These full text articles were read, and after application of selection criteria, there were 59 articles chosen for final review (see Figure 1). Fifty-five articles were from Pubmed, and four articles from reference lists.

The initial search was limited to articles published in English. The articles selected for final review examined populations in sub-Saharan Africa, were original studies (not review articles), and were either specific to bed nets and pregnant or post-partum women, or were studies that extracted data specific to pregnant or post-partum women from wider household or national data sets. Articles were excluded if they primarily concerned the economic aspects of ITN distribution or efficacy of ITNs, as these topics were outside the scope of this review. Articles reporting qualitative studies were excluded, as there had been a recently published systematic review of qualitative data regarding malaria prevention interventions in pregnancy [11].

Articles were classified based on year of publication, country of study, type of study, sample size, urban/ rural/mixed residence, location of study (household/ health facility/ANC) and whether the study concerned pregnant women alone, or a mixed population. Selected articles were saved in Endnote® and their characteristics manually entered into Microsoft Excel for ongoing data management. From these articles, data corresponding to the definitions, above, was extracted.

## Results

### Characteristics of studies

**Figure 2:**
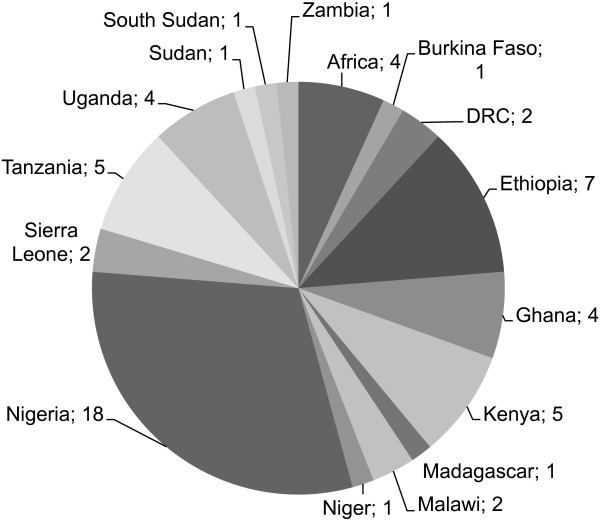
Country where studies were undertaken.

Of the 59 articles selected for review, almost one third of the data came from Nigeria (30.5%), with multiple contributions from Ethiopia, Kenya, Tanzania and Ghana (see Figure 2).

There were 29 articles specifically concerning pregnant women, and 30 articles with household data from which information regarding pregnant women had been extracted. Eighty-six percent of articles reported studies that were cross-sectional in nature, with 8% longitudinal, and one case control, one cohort and one cluster randomized control trial. Sixty-four percent of data came from household surveys, and 34% from surveys conducted at antenatal clinics or at hospitals. Residence of study subjects was largely mixed between rural and urban (49%), with the remaining studies concentrating on rural subjects alone (30%) or urban subjects (20%).

The articles selected contained a mix of routine ITN coverage data from DHS and MIS for mixed populations and pregnant women at national and district levels, as well as contextually specific data regarding ownership and use following the implementation of a specific programme or intervention as well as Knowledge Attitude Practice studies of households and pregnant women attending health facilities or ANCs. The articles selected for review, and the type of information they provided, are summarized in Additional file 1.

The Malaria Indicator Survey differentiates between non-treated, treated and LLIN bed nets. Most of the articles reviewed concerned insecticide-treated nets, and where this was not specified, it was assumed that the nets were insecticide treated, as this is the most common net currently being distributed. If there was specific data pertaining to untreated nets, this was not included in data presented.

### ITN coverage and available ITN use

A wide variation in the ITN coverage rates found in sub-Saharan Africa has been reported, however the general trend showed improvements in both ownership and use of ITNs amongst pregnant women in the last decade [9,10]. In households containing pregnant women, ownership ranged from 3 to 44%, in a summary article of data from 2003 to 2007 from 15 African nations [10].

As per the analysis of Van Eijk *et al.*[9], the poorest performing countries at a national level, in terms of overall coverage (ITN use the night prior), were Swaziland 1% (DHS 2006–07), Nigeria 5% (2008 DHS) and Zimbabwe 6% (2007 MIS). The best performing countries or regions were Zanzibar 51%, Tanzania 26% (MIS 2007–08), Kenya 49% (2008–09 DHS) and Madagascar 46% (2008–09 DHS). A few declining trends were also observed. In Ethiopia overall household ownership of ITNs reduced from 66% in the 2007 MIS [16] to 49% in a 2009 HH survey [17], which also corresponded to an overall decline in available ITN use from 84% in 2006 to 59% in 2009, although this was highly variable within different regions. Between 2000 and 2004, there was a reported decline in ITN use from 58 to 46% in Nigeria, despite an increase in ownership rates (statistical significance of this difference was not calculated), representing a large increase in unused bed nets [18]. The surveys had been conducted in the same districts and in the same seasons, and the authors were unable to account for this decrease. Unfortunately they did not explore whether the deterioration of pre-existing ITNs could have contributed to this result.

**Figure 3:**
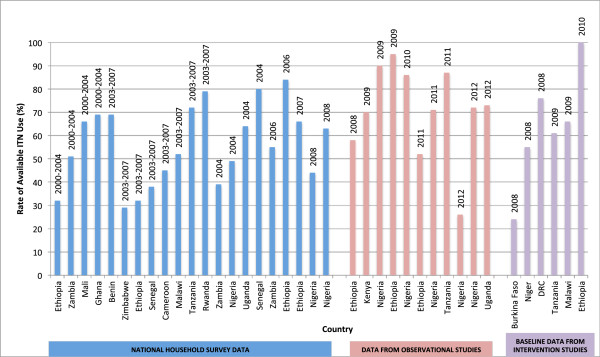
**Reported rates of available ITN use by pregnant women (ownership 100%).** Each bar represents reported rates of ITN use by pregnant women in households owning at least one ITN, from the specified country and year. National survey data have been presented by year of survey, whereas data from observational and intervention studies have been presented by the year of publication. Where a range for ITN use was reported (e.g., between districts), the higher rate has been charted.

Fourteen of the articles directly measured pregnant women’s use of available ITNs (where there is at least one ITN in the household), and this statistic was able to be calculated from a further 11 studies. The details of these are presented in Figure 3 and Additional file 2, which stratifies data into national household surveys, observational studies and baseline data from intervention studies. In general, national data showed lower rates of available use by pregnant women, whereas studies conducted at health facilities showed higher rates of available ITN use and coverage. There were only two household surveys targeting pregnant women specifically. One of these, a large study from Nigeria (n= 2,348), revealed low rates of available ITN use (26%), when compared with DHS data and sampling from women attending ANC [19]. This reflects the wide variation that can occur even within a country, and the biases that occur between the various methods of data collection. Specific intervention studies, as expected, showed higher rates of ITN use and coverage. In Uganda and Tanzania there was less discrepancy between ownership and use of ITNs, meaning that 67-89% of pregnant women were protected by ITNs.

### Determinants of ITN ownership and use

Forty-four of the selected articles made comment on factors influencing ownership and/or use of ITNs amongst pregnant women. The statistically significant determinants are presented in Additional file 3. Factors significantly associated with ownership included: education, knowledge of malaria or ITNs, and marital status. Factors associated with use included: education, household income, socio-economic status or ownership of goods, malaria and ITN knowledge, and urban residence. In the larger studies where sample size exceeded 1,000 persons, factors found to be significantly associated with use included: wealth, urban residence, malaria or ITN knowledge, lack of misconceptions, older age (variable comparison groups between studies), and number of ITNs in the household [17,19-22]. Whether these factors were statistically significantly associated with ITN use varied considerably by study, independent of sample size, and the direction of the association was not consistent between countries.

Paradoxically, in two large household studies conducted in Uganda and Nigeria, a lower education was associated with significantly higher rates of ITN use amongst pregnant women [20,21]. This was postulated by Auta *et al.*[21] to be related to an increased perceived vulnerability to malaria in poorer households. The study found that the lowest wealth quintile also demonstrated higher rates of ITN use (OR 2.3, 95% CI 1.1-5). This finding could also be the result of targeted public health campaigns.

Stock-out (or unavailability), cost, and failure to issue vouchers were frequently identified as barriers to ownership [23-25]. Cost was identified as a barrier to ownership even in settings where subsidies via voucher schemes were available. Regarding failure to use available ITNs, reported reasons included discomfort, heat or inconvenience, limited perceived benefit of ITNs, and preference to use other malaria prevention methods. A summary of these barriers is presented in Figure 1. As this data came from disparate sources, it was difficult to determine the scale and importance of each of these factors, which are likely to be context specific.

### Specific interventions

Where specific interventions were evaluated for their effect on ownership and use, they have been presented in Additional file 4 (n=19). Studies looking at the effect of national campaigns using DHS data were not included in this part of the review, only those that evaluated a particular strategy or aspect of distribution.

Nine studies looked at ITN distribution in which a behavioural or educational component was a key part of the intervention. These included a variety of techniques including demonstrations by volunteers, information leaflets, social marketing and ‘hang up’ campaigns, and provision of materials and tools. All the studies identified showed considerable gains in terms of increasing ownership, use and retention in the target groups, except for one programme in Uganda, where both ownership and use remained low despite an intervention for distributing subsidized ITNs with education. The authors of that study concluded that the programme had been inequitable and that the information delivered was inadequate [20]. The successful programmes saw increases (pre- to post-intervention) in ITN coverage from 25 to 80% in the Democratic Republic of Congo [26], 50 to 100% in Ghana [27], 37 to 87% in Sierra Leone [28] and 55 to 63% in Tanzania [29]. Interestingly, only one article made mention of the role of social marketing in improving ITN ownership and use amongst pregnant women [30]; this study saw increases in ownership of ITNs from 13 to 35% in Burkina Faso. It would appear that the improvements in coverage rates were not only due to increased ownership, but also due to increases in available ITN use (where this was specifically measured).

Five articles evaluated voucher schemes for ITNs at ANCs, two from Ghana and three from Tanzania. Another study evaluated a voucher scheme run through household distribution and pick-up points in Sierra Leone [28]. All studies highlighted the several steps involved in achieving targets, from voucher distribution, offering, acceptance, redemption and ownership, to ITN use. The cumulative effect of the voucher schemes in the various settings appeared to be hampered by inadequate issuing of vouchers, and failure of recipients to redeem the given vouchers. Failure to issue vouchers was most often related to perceived or actual ineligibility or client refusal. In some instances vouchers were not distributed if health workers assumed that potential recipients would be unable to afford even the subsidized ITN [23].

Another five articles assessed free ANC distribution of ITNs (n=3) and integrated campaigns with other interventions such as childhood vaccination (n=2), all of which were successful in increasing ITN ownership. In the Ugandan example, routine free ITNs at ANCs were compared with two once-off mass campaigns for ITN distribution at a household level. Ownership increased to greater than 90% in all test groups, with high rates of retention, and use was highest amongst women receiving ITNs via ANC (99%) compared with 74 and 97% in the test groups [31].

## Discussion

The last five years have seen significant gains made in sub-Saharan Africa in terms of scaling up coverage of ITNs in pregnancy, especially in light of supportive policies and financial investments from national governments, but there remains a wide range in the coverage rates at both national and subnational levels.

This review highlights the disappointing discrepancy between ownership and use of ITNs amongst pregnant women in many settings, representing a missed opportunity for distribution programmes to address the reasons for this trend. Bed net use cannot simply be assumed in households receiving and owning ITNs. Available ITN use by pregnant women was as high as 82% in Kenya, and as low as 29% in Zimbabwe, in national data sets. Cited reasons for this discrepancy included non-modifiable factors, such as discomfort and inconvenience, but interestingly issues of knowledge and perceived vulnerability to malaria were also frequently mentioned. Available ITN use should be measured more frequently as an important part of programme evaluations, and where low, the underlying reasons should be explored further.

Some of the aforementioned barriers to ITN use could be overcome by including education at the time of distribution. Unfortunately there were no comparative studies that examined programmes with and without an educational component, but where an educational or marketing campaign was employed as part of an ITN delivery programme there appeared to be encouraging results, with coverage rates increasing to greater than 70% post intervention [26,27,32,33]. Additionally one cluster study from Nigeria showed that a community designed and implemented ITN distribution and educational programme was more successful in increasing coverage (by an additional 7-9%) than routine national ANC distribution [34]. This type of innovative participatory approach to engage communities and facilitate behaviour change has been used successfully for other public health interventions, and may be useful in settings where ITN coverage remains low. Other interventions such as hang up campaigns, provision of tools and use of community volunteers for home visits demonstrated how some of the physical barriers to use, such as problems with hanging or incorrect use could be overcome.

At the individual level, ownership rates ranged from 8-90% in larger household and surveillance data sets, and from 9-100% in smaller studies. Similarly, available ITN use ranged from 29-84% to 26-100%. In general, smaller studies showed higher rates of ownership and usage, particularly where a specific intervention was employed. Despite this, overall it would appear that only half of pregnancies in sub-Saharan Africa are covered by this effective intervention, with ownership being influenced by delivery systems, areas of residence and attendance at antenatal clinics.

At the supply level, unpredictable supply and frequent stock-outs were commonly reported in the literature as a barrier to ownership. In settings where voucher schemes had been adopted, there were additional potential bottlenecks reported, including the supply of vouchers, health personnel’s attitudes towards distributing vouchers, customer acceptance and redemption of vouchers, and stock-outs of ITN supply at a market level. In both Ghana and Tanzania, a considerable drop in the cumulative success of the voucher schemes was attributable to both stock-outs and failure to issue ITN vouchers to women who were eligible to receive them, possibly because the health worker responsible for distributing vouchers did not think the pregnant woman would be able to afford the required financial contribution [23,25]. This highlights the need for adequate training of health care workers and attention to all building blocks of the health care system in delivering interventions.

**Figure 4:**
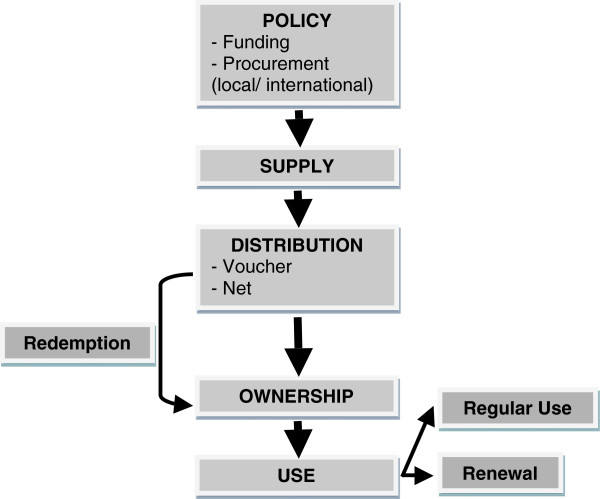
Steps in achieving ITN coverage in pregnancy.

There are several steps involved in achieving adequate ITN coverage (see Figure 4), and the sequential steps between each process represent potential bottlenecks if the health system is to deliver the intended benefits. The barriers to achieving adequate ITN coverage (see list of ‘Reported Barriers’ section) should be considered prior to implementing and running a malaria intervention programme, to ensure its success. The high level of variability found throughout sub-Saharan Africa, in terms of determinants of, and barriers to, ownership and use, as well as the successes of different interventions, suggests that context specific responses are required, and that community participation can perhaps increase the chances of a successful programme. The Nigerian and Ethiopian examples of declining ITN ownership post mass distribution campaigns serve as reminders that interventions must be maintained, and ongoing supply ensured to keep up progress that has been made initially [17,18].

### Reported barriers to ownership and use of ITNs amongst pregnant women (Presented in decreasing frequency of being reported)

Supply/ Distribution

○ Stock out of ITN or vouchers

○ Failure to issue voucher to eligible clients

○ Failure to redeem vouchers

○ Lack of ANC attendance

○ Refusal of coupon

○ Lost coupon

Ownership

○ Cost

○ No belief in ITN as prevention method

○ Other methods preferred (sprays, window nets)

○ Did not know where to get

○ Wanted to have own choice in net

○ Did not like ITNs

○ Increased distance from ANC

○ Felt that bought ITNs were better

Use

○ Discomfort (heat/ sweat/ breathing)

○ Not enough room/ place to tie/ burdensome to tie

○ Low awareness of need

○ Misuse (hung over windows, used as blanket)

○ Low awareness of how to use

○ Low mosquito activity/ seasonal

○ Someone else in family using net (children mostly)

○ No belief in protection method

○ Mosquitoes still bite

○ Saving use for post partum period

○ Net too small for family

Maintenance

○ Stolen/ damaged

○ Not retreating nets

The findings in this review echo and add to the information from Pell’s review, which was confined to qualitative studies, of factors influencing women’s uptake of malaria interventions in Africa [11]. Recurring themes include perceived risk, malaria beliefs and knowledge, seasonality, ITN inconvenience and health worker attitudes.

Measuring the uptake of malaria interventions in pregnant women, and particularly ITN use, is fraught with many difficulties. First, the coverage indicator for ITN use (use of ITN the night prior) is based on self-reporting and liable to information bias, such as overreporting by women, especially post partum women asked to recall their use habits during pregnancy, who may feel obliged to respond in a certain way. Behaviour may also be subject to seasonal variation, a particular problem for cross-sectional studies. Also lacking is the differentiation between overall use of nets, and the use of available nets, which would help define whether low use of bed nets amongst pregnant women is behavioural or related to a supply issue.

Additionally, adequate coverage in pregnancy is not well defined. Evidence suggests that malaria prevention is likely to have benefit from very early in pregnancy, before the first ANC visit, which is often after 20 weeks [35]. There was a paucity of data concerning the timing of implementation of the intervention, or what proportion of an individual pregnancy was protected by ITNs. This important information gap could be examined in future studies.

Evidence-based rationalization of ITN coverage targets is needed, but more research is required into setting-specific vector behaviour and malaria transmission trends, to predict what levels of community coverage might provide protection to all members, including those not sleeping under a net. Until then, targeting those most at risk (such as pregnant women and children) would be appropriate. Encouragingly, Baume *et al.*[18] looked at intra-household net use, in households containing nets in six different sub-Saharan countries, to determine who was most likely to use nets and found that younger children and pregnant women were more likely to sleep under a net, in net owning households (compared with men and older children). A few other studies also found that pregnant women were more likely to use bed nets in households owning more than one bed net [17,26,28]. It would have been interesting to be able to take into account the likely non-random distribution of nets and where in the hierarchy a pregnant woman stood when insufficient nets were available for all members of a household.

In the studies reviewed here, sampling bias was evident. In particular, most studies that focused on pregnant women were carried out at a health facility, which does not adequately sample the women who do not attend health care facilities for antenatal care, and may only capture women later in their pregnancies. The gaps in coverage during early pregnancy, and of women who do not attend health care facilities for antenatal care, could be addressed with improvements in community level interventions.

## Conclusion

This review identifies several gaps in health systems in delivering successful ITN distribution programmes, as well as highlighting issues at an individual level that prevent pregnant women from using the bed nets that they own. Cost, stock-outs and failed distribution and redemption of vouchers were found to be barriers to ownership, and inconvenience and lack of knowledge about ITNs and malaria were frequently cited as reasons for not using nets.

The combination of low ownership rates and available nets not being used resulted in disappointing coverage rates, leaving more than half of all pregnancies at risk of malaria in several settings. Encouragingly, there were some successful national programmes, such as the voucher system in Tanzania, as well as some innovative regional interventions, which employed the use of additional education, marketing and community participation. These provide examples of how some of the barriers to ownership and use can be overcome.

Future research needs to be directed at clarifying the relationship between ITN ownership and use in pregnancy (by specifically measuring indicators for available ITN use, in households owning at least one bed net), as well as rationalising coverage targets to achieve desired the effects in terms of reduced disease burden. Consideration could be given to targeting all women of reproductive age, to ensure optimal coverage for the duration of an entire pregnancy. Health systems need to reflect on the contextual barriers to ownership and use when planning ITN distribution programmes, with attention to funding, procurement, staff training and sustainability. Consideration should be given to how community involvement and educational components may have a greater role in scaling up ITN interventions, particularly in settings where coverage rates are low.

## Abbreviations

ANC: Antenatal clinic; DHS: Demographic and health survey; ITN: Insecticide-treated net; LLIN: Long-lasting insecticide net; MIS: Malaria indicator survey; RBM: Roll Back Malaria; WHO: World Health Organization; IPT: Intermittent preventive treatment.

## Competing interests

The authors have declared that they have no competing interests.

## Authors’ contributions

MS, GB and SR developed the review topic and objectives. MS conducted the literature review and drafted the initial manuscript. All authors contributed to the review and production of the final manuscript.

## Supplementary Material

Additional file 1 **Summary of the 59 articles selected for review (organized by country) **[36]**-**[72]**.** Click here for file

Additional file 2 Use of available ITNs by pregnant women. Click here for file

Additional file 3 Determinants of ITN ownership and use in pregnancy (arranged by country). Click here for file

Additional file 4 Interventions to improve ITN coverage. Click here for file
