# Supplementary material for: Ownership and use of insecticide-treated nets during pregnancy in sub-Saharan Africa: a review
Source: Malar J. 2013 Aug 1;12:268. doi: 10.1186/1475-2875-12-268 (PMC3734149; doi:10.1186/1475-2875-12-268)
Supplement: Additional file 1 — Summary of the 59 articles selected for review (organized by country) [36]-[72]. [file 1475-2875-12-268-S1.pdf]

Additional File 1: Summary of the 59 articles selected for review (organized by country)

	STUDY CHARACTERISTICS							RELEVANT INFORMATION CONTAINED			
**Author**	**Year**	**Country**	**Type**	**Sample size**	**Study setting**	**Rural/ Urban/ Mixed**	**Study Pop**	**Routine Coverage**	**Available ITN Use**	**Ownership/ use determinants**	**Specific Interventions**
**van Eijk *et al* [9]**	**2011**	**Africa**	Cross section	N/A	HH	M	Mixed	✓			
**Eisele *et al* [10]**	**2009**	**Africa**	Cross section	N/A	HH	M	Mixed	✓	✓		
**Baume *et al* [36]**	**2008**	**Africa**	Cross section	N/A	HH	M	Mixed	✓	✓		
**Baume *et al* [18]**	**2007**	**Africa**	Cross section	N/A	HH	M	Mixed		✓		
**Muller *et al* [30]**	**2008**	**Burkina Faso**	Cluster RCT	72/107p 100/105p	HH	R	Pregnant		✓		✓
**Pettifor *et al* [26]**	**2009**	**DRC**	Longitudinal	362p 328p	ANC	U	Pregnant	✓		✓	✓
**Pettifor *et al* [37]**	**2008**	**DRC**	Cross section	351p	ANC	U	Pregnant	✓	✓	✓	
**Deressa *et al* [17]**	**2011**	**Ethiopia**	Cross section	2874HH	HH	R	Mixed	✓	✓	✓	
**Graves *et al* [16]**	**2011**	**Ethiopia**	Longitudinal	240p 232p	HH	M	Mixed		✓		
**Deribew et al[22]**	**2010**	**Ethiopia**	Cross section	4135 HH 242p	HH	M	Mixed		✓	✓	
**Jima *et al* [38]**	**2010**	**Ethiopia**	Cross section	32380 570p	HH	M	Mixed	✓	✓		
**Karunamoorthi *et al* [39]**	**2010**	**Ethiopia**	Cross section	225p	ANC	U	Pregnant			✓	
**Graves *et al* [40]**	**2009**	**Ethiopia**	Cross section	2549 207p	HH	M	Mixed	✓	✓		
**Belay *et al* [41]**	**2008**	**Ethiopia**	Cross section	815p	HH	R	Pregnant		✓	✓	
**Rickard *et al* [27]**	**2011**	**Ghana**	Longitudinal	13p	HH	R	Mixed			✓	✓
**Odouro *et al* [42]**	**2010**	**Ghana**	Cross section	2232p	ANC	R	Pregnant			✓	
**Webster *et al* [43]**	**2010**	**Ghana**	Longitudinal	1232p 1226p	HH	M	Pregnant			✓	✓
**Keweku *et al* [23]**	**2007**	**Ghana**	Cross section	25926p	ANC	M	Pregnant			✓	✓
**O'Meara *et al* [44]**	**2011**	**Kenya**	Cross section	44753HH 2988p	HH	R	Mixed			✓	
**Hightower *et al* [45]**	**2010**	**Kenya**	Cross section	182p	HH	M	Mixed			✓	✓
**Njoroge *et al* [46]**	**2009**	**Kenya**	Cross section	220p	ANC	R	Pregnant		✓		
**Gikandi *et al* [47]**	**2008**	**Kenya**	Cross section	976p	HH	R	Pregnant			✓	
**Ouma *et al* [48]**	**2007**	**Kenya**	Cross section	685p	ANC	U	Pregnant			✓	
**Kulkarni *et al* [49]**	**2010**	**Madagas-car**	Cross section	2756 320 p	HH	M	Mixed				✓
**Skarbinski *et al* [50]**	**2011**	**Malawi**	Cross section	634p	HH	M	Mixed				✓
**Mathanga *et al* [51]**	**2009**	**Malawi**	Longitudinal	78p 149p	HH	R	Mixed		✓		✓
**Thwing *et al* [32]**	**2008**	**Niger**	Longitudinal	254p 328p	HH	M	Mixed		✓	✓	✓
**Mbachu *et al* [52]**	**2012**	**Nigeria**	Cross section	2394 HH 898p	HH	M	Mixed	✓		✓	
**Ordinioha [53]**	**2012**	**Nigeria**	Cross section	170 19p	HH	M	Mixed	✓		✓	
**Aluko *et al* [54]**	**2012**	**Nigeria**	Cross section	335p	HH	U	Pregnant		✓	✓	

Author	Year	Country	Type	Sample size	Study setting	Rural/ urban/ mixed	Study Pop	Routine Coverage	Available ITN Use	Ownership/ use determinants	Specific Interventions
**Ankomah *et al* [19]**	**2012**	**Nigeria**	Cross section	2348p	HH	M	Pregnant		✓	✓	
**Auta *et al* [21]**	**2012**	**Nigeria**	Cross section	34070 HH	HH	M	Mixed		✓	✓	
**Aina *et al* [72]**	**2011**	**Nigeria**	Cross section	163p	ANC	U	Pregnant		✓	✓	
**Akinleye *et al* [55]**	**2011**	**Nigeria**	Cross section	209p	ANC	R	Pregnant			✓	
**Amzat *et al* [56]**	**2011**	**Nigeria**	Cross section	34070 HH	HH	M	Mixed	✓	✓		
**Okeibunor *et al* [34]**	**2011**	**Nigeria**	Case control	1280p 1380p	HH	M	Pregnant				✓
**Tongo *et al* [57]**	**2011**	**Nigeria**	Cross section	796p	HF	U	Pregnant			✓	
**Wagbatsoma *et al* [58]**	**2010**	**Nigeria**	Cross section	385p	ANC	R	Pregnant		✓	✓	
**Afolabi *et al* [59]**	**2009**	**Nigeria**	Cross section	439 78p	HH	R	Mixed		✓	✓	
**Isah et al [60]**	**2009**	**Nigeria**	Cross section	250p	ANC	U	Pregnant			✓	
**Musa et al [2]**	**2009**	**Nigeria**	Cross section	455p	ANC	R	Pregnant			✓	
**Sam - Wobo et al [61]**	**2008**	**Nigeria**	Cross section	1400p	HH	M	Pregnant			✓	
**Yusuf et al [62]**	**2008**	**Nigeria**	Cross section	983p	HF	U	Pregnant			✓	
**Adeneye et al [63]**	**2007**	**Nigeria**	Cross section	68p	ANC	R	Mixed			✓	
**Enato *et al* [64]**	**2007**	**Nigeria**	Cross section	867p	ANC	U	Pregnant			✓	
**Bennett *et al* [28]**	**2012**	**Sierra Leone**	Cross section	4620 HH 609p	HH	M	Mixed			✓	✓
**Gerstl *et al* [33]**	**2010**	**Sierra Leone**	Cross section	137p	HH	M	Mixed			✓	✓
**Napoleon *et al* [65]**	**2011**	**South Sudan**	Cross section	334p	ANC	U	Pregnant			✓	
**Hassan *et al* [66]**	**2012**	**Sudan**	Cross section	19p	HH	M	Mixed	✓		✓	
**West *et al* [29]**	**2012**	**Tanzania**	Cross section	228p 224p	HH	R	Mixed	✓		✓	✓
**Ambrose *et al* [67]**	**2011**	**Tanzania**	Cross section	222p	ANC	U	Pregnant		✓	✓	
**Marchant *et al* [68]**	**2011**	**Tanzania**	Cross section	363p- 915p	ANC	M	Pregnant			✓	✓
**Marchant *et al* [24]**	**2010**	**Tanzania**	Cross section	2027p 707p	HH	M	Pregnant			✓	✓
**Hanson *et al* [69]**	**2009**	**Tanzania**	Cross section	584p- 779p	HH	M	Mixed	✓	✓	✓	✓
**Sangare *et al* [70]**	**2012**	**Uganda**	Cross section	500p	HH	R	Pregnant	✓		✓	
**Ahmed *et al* [20]**	**2010**	**Uganda**	Cross section	10234 HH	HH	M	Mixed			✓	✓
**Kolaczinski *et al* [31]**	**2010**	**Uganda**	Cross section	328p	HH	M	Mixed			✓	✓
**Kiwuwa *et al* [35]**	**2008**	**Uganda**	Cross section	769p	HH	R	Pregnant			✓	
**Steketee *et al* [71]**	**2008**	**Zambia**	Cross section	2999 HH	HH	M	Mixed	✓	✓		

Legend: p Pregnant, HH Household, HF Health Facility, ANC Antenatal Clinic, Pop Population, Mixed Study examined a general population, R Rural, U Urban
