# Supplementary material for: Ownership and use of insecticide-treated nets during pregnancy in sub-Saharan Africa: a review
Source: Malar J. 2013 Aug 1;12:268. doi: 10.1186/1475-2875-12-268 (PMC3734149; doi:10.1186/1475-2875-12-268)
Supplement: Additional file 2 — Use of available ITNs by pregnant women. [file 1475-2875-12-268-S2.pdf]

Additional File 2: Use of available ITNs by pregnant women

Author	Year	Country	Type	n=	Data Source	ITN OWNERSHIP (at least 1 ITN/HH)	AVAILABLE ITN USE (use in HH owning >/= 1 ITN)	Coverage *
**NATIONAL HOUSEHOLD SURVEY DATA**								
**Eisele *et al* [10]**	**2009**	**Africa**	Cross section	N/A	DHS and MIS data 2003-2007 from 15 Nations.	100% HH (for analysis)	Kenya 82%, DRC 73%, Rwanda 79%, Tanzania 72%, Zimbabwe 29%, Ethiopia 32%, Senegal 38%, Malawi 52%	
**Baume *et al* [36]**	**2008**	**Africa**	Cross section	N/A	Coverage of ITNs from 2000-2004 from HH surveys in 4 nations.	Nigeria 0-9%<br>Senegal 8-39%<br>Zambia 6-35%<br>Uganda 1-21%	Nigeria 49% *(2004)<br>Senegal 80% *<br>Zambia 39% *<br>Uganda 64% *	Nigeria 0-4%<br>Senegal 5-31%<br>Zambia 0-14%<br>Uganda 1-13%
**Baume *et al*[18]**	**2007**	**Africa**	Cross section	N/A	Coverage of ITNs from 2000-2004 from DHS in 6 Nations.	100% HH (for analysis)	Ghana 69%, Mali 66%, Zambia 51%, Ethiopia 32%	
**Graves *et al*[16]**	**2011**	**Ethiopia**	Longitudinal	240p<br>232p	Data from 2006 MIS and sample of HH in 2007.	100% HH (for analysis)	84% (2006)<br>59% (2007)	
**Jima *et al*[38]**	**2010**	**Ethiopia**	Cross section	570p	2007 MIS Results.	66% HH	66%	44%
**Auta *et al*[21]**	**2012**	**Nigeria**	Cross section		2008 DHS.	8% HH	44%	4%
**Amzat *et al*[56]**	**2011**	**Nigeria**	Cross section		2008 DHS + other data	8%	63% *	5%
**Steketee *et al*[71]**	**2008**	**Zambia**	Cross section		2006 DHS	44%	55% *	24%
**DATA FROM OBSERVATIONAL STUDIES**								
**Deressa *et al*[17]**	**2011**	**Ethiopia**	Cross section	2874	HH survey in 2 districts.	49% HH	52%	25%
**Graves *et al*[40]**	**2009**	**Ethiopia**	Cross section	207p	Modified MIS in from 3 districts.	20% in HH	95% *	19%
**Belay *et al*[41]**	**2008**	**Ethiopia**	Cross section	815p	Community based HH survey.	34% HH	58%	20%
**Njoroge *et al*[46]**	**2009**	**Kenya**	Cross section	220p	Survey of women attending ANC.	75%	70%	53%
**Aluko *et al*[54]**	**2012**	**Nigeria**	Cross section	335p	Surveys of women at fee-paying hospitals.	44%	72%	32%
**Ankomah *et al*[19]**	**2012**	**Nigeria**	Cross section	2348p	HH surveys of pregnant women.	29%	26%	8%
**Aina *et al*[72]**	**2011**	**Nigeria**	Cross section	163p	Survey of women attending ANC.	16-55 %	68-71%	11-37%
**Wagbatsoma *et al*[58]**	**2010**	**Nigeria**	Cross section	385p	Survey of women attending ANC.	9% HH	86%	8%
**Afolabi *et al*[59]**	**2009**	**Nigeria**	Cross section	78p	HH survey in 2 rural regions.	22-49%	58-90% *	29-28%
**Ambrose *et al*[67]**	**2011**	**Tanzania**	Cross section	222p	Survey of women attending ANC.	78%	87%	68%
**Sangare *et al*[70]**	**2012**	**Uganda**	Cross section	500p	HH survey of pregnant/ post partum women.	72% 1st TM<br>+20% 2nd TM	73%	67%
**BASELINE DATA FROM INTERVENTION STUDIES**								
**Muller *et al*[30]**	**2008**	**Burkina Faso**	Cluster RCT	107p<br>105p	Baseline data from 2 rural clusters in a RCT.	21-25%	20-24% *	5% (both clusters)
**Pettifor *et al*[37]**	**2008**	**DRC**	Longitudinal	351p	Baseline data from a wider longitudinal study.	33%	76% *	25%
**Deribew *et al*[22]**	**2010**	**Ethiopia**	Cross section	242p	Baseline results from a cluster RCT.	70%	80%-100% *	56- 75%
**Mathanga *et al*[51]**	**2009**	**Malawi**	Longitudinal	78p<br>149p	Baseline data from a rural intervention study.	41-57%	49-66% *	22-29%
**Thwing *et al*[32]**	**2008**	**Niger**	Longitudinal	254p	Baseline data from a campaign evaluation.	6%	55% *	31%
**Hanson *et al*[69]**	**2009**	**Tanzania**	Cross section	779p	Baseline data from a voucher evaluation.	18%	61% *	11%

Legend: NA not available, DRC Democratic Republic of Congo, HH Household, p pregnant, MIS Malaria Indicator survey, TM trimester, *RCT Randomized Control Trial*. Where statistics have been presented, all have been rounded to the nearest whole number.
*Calculated from available data using formula [% coverage= % ownership x % available ITN use]
