# Supplementary material for: Ownership and use of insecticide-treated nets during pregnancy in sub-Saharan Africa: a review
Source: Malar J. 2013 Aug 1;12:268. doi: 10.1186/1475-2875-12-268 (PMC3734149; doi:10.1186/1475-2875-12-268)
Supplement: Additional file 3 — Determinants of ITN ownership and use in pregnancy (arranged by country). [file 1475-2875-12-268-S3.pdf]

Additional File 3: Determinants of ITN ownership and use in pregnancy (arranged by country)

Author	Year	Country	n=	Where	Study Summary	Factors significantly associated with ITN ownership/ use (OR/CIs if provided)	Analysis
**Pettifor *et al* [37]**	**2008**	**DRC**	351p	ANC	Baseline data from a wider longitudinal study. Bed net ownership, use and perceptions among women seeking ANC.	***Ownership:*** >/= Secondary education vs. less education OR 3.4 (1.6-7.3), ***Use:*** Education OR 2.8 (1.3-6.0)	MV
**Deressa *et al* [17]**	**2011**	**Ethiopia**	2874HH	HH	2009 HH survey in 2 districts, 2 years after a free distribution campaign.	***Use:***>1 ITN in the household OR 2.9 (2.2-3.9), ITN knowledge OR 1.5 (1-2.3), lack of problems in using ITNs OR 1.9 (1-3.6)	MV
**Deribew *et al* [22]**	**2010**	**Ethiopia**	4135 HH 242p	HH	HH survey. Baseline results of a cluster randomized trial assessing LLINs distribution program.	***Use:*** Malaria knowledge OR 2.0 (1.4-2.8)	MV
**Karunamoorthi *et al* [39]**	**2010**	**Ethiopia**	225p	ANC	Survey of women attending ANC.	***Use:*** Women's primary education vs. illiteracy (p<0.001)	Chi
**Belay *et al* [41]**	**2008**	**Ethiopia**	815p	HH	HH survey.	***Use***= Education (literate or not) OR 3.1 (2.1-4.6)	MV
**Odouro *et al* [42]**	**2010**	**Ghana**	2232p	ANC	Baseline data presented here from prospective cohort study.	***Use:*** Older age, more years of education, higher ANC clinic attendance, better IPT coverage, history of miscarriage or the death of a child	UV
**O'Meara *et al* [44]**	**2011**	**Kenya**	44753 HH 2988p	HH	Data from census to examine the extent of, and factors correlated with, ownership of bed nets.	***Ownership:*** Urban HH animal ownership OR 1.5 (1.2-1.8) Urban nearest facility hospital OR 2.9 (1.3-6.6), Rural nearest facility health centre OR 1.2 (1.1-1.4)	MV
**Hightower *et al* [45]**	**2010**	**Kenya**	182p	HH	HH surveys relating to use of ITN and ownership in children under 5 and women of reproductive age. Conducted one month after a free distribution campaign.	***Ownership:*** Urban residence OR 1.4 (1.1-1.8) ***Use:*** Wealth (OR not provided)	UV
**Gikandi *et al* [47]**	**2008**	**Kenya**	976	HH	Examining barriers to ITN/ IPT.	***Use:*** IPT use OR 1.4 (1-1.8), wealth OR 2.5 (1.4-4.7), multi-gravid OR 1.5 (1-2.4)	MV
**Ouma *et al* [48]**	**2007**	**Kenya**	685	ANC	Malaria and anaemia among pregnant women at first ANC visit.	***Use:*** Marital status single RR 0.7 (0.5-1), Education <8 yrs RR 0.7 (0.5-0.9), Electricity in HH RR 1.5 (1.2-2), Full time employment RR 1.3 (1-1.6), Listening to radio 1/ wk RR 1.2 (1-1.5)	MV
**Aluko *et al* [54]**	**2012**	**Nigeria**	335	Hosp.	Utilisation of ITN in pregnancy amongst post partum women.	***Ownership:*** Years of education, monogamous families, employment, residence in low population density area, religion (Christian) ***Use:*** Monogamous families, Education, Woman dependent (not employed)	MV
**Ankomah *et al* [19]**	**2012**	**Nigeria**	2348	HH	Determinants of ITN ownership and utilization.	***Ownership:*** ITN Knowledge OR 3.9 (3-4.8), ANC Registration OR 1.3 (1.1-1.6), Urban residence OR 0.8 (0.7-1) ***Use:*** Urban residence OR 1.9 (1.3-2.7), ITN Knowledge OR 2.9 (1.9-4.6), No misconceptions OR 1.6 (1.1-2.3)	MV
**Auta *et al* [21]**	**2012**	**Nigeria**	34070 HH	HH	Demographic factors associated with the use of ITNs among women and pregnant children.	***Use:*** Without formal education OR 2.7 (1.1-6.6), Lowest wealth quintile OR 2.3 (1.1-5)	UV
**Akinleye *et al* [55]**	**2011**	**Nigeria**	209p	Hosp.	Determining the level of knowledge of malaria and related preventive measures.	***Use:*** ITN knowledge OR 0.9 (1-5.8)	BV

Author	Year	Country	n=	Where	Study Summary	Factors significantly associated with ITN ownership/ use (OR/CIs if provided)	Analysis
**Wagbatsoma *et al* [58]**	**2010**	**Nigeria**	385p	ANC	ITN utilization amongst those women attending ANC.	***Use:*** Small family size	Fisher's
**Bennett *et al* [28]**	**2012**	**Sierra Leone**	4620 HH 609p	HH	National survey 6 months post LLIN distribution campaign (universal coverage aim) + hang up campaign.	***Use:*** Older age 35-49 yo vs 15-24 yo OR 6.1 (1.9-19.3), having a hanging ITN, >1 ITN/ 2ppl in HH	MV
**Napoleon *et al* [65]**	**2011**	**South Sudan**	334p	ANC	Cross sectional survey at a tertiary hospital.	***Use:*** Having bought an ITN OR 504.1 (91.5-2777.9), use of indoor spray OR 16.6 (1.3-206.2), higher HH income OR 0.17 (0.04-0.7)	MV
**Hassan *et al* [66]**	**2008**	**Sudan**	19p	HH	HH survey 1 year post distribution, looking at retention and use of ITNs.	***Ownership:*** Lower education, more nets<br>***Retention:*** Higher in those with formal education<br>***Use:*** higher in those who received ITN education	Chi
**Ambrose *et al* [67]**	**2011**	**Tanzania**	222p	ANC	Knowledge Attitude Practice surveys regarding malaria/ bed net use amongst women attending ANC.	***Ownership:*** Multigravid OR 2.1 (1.2-4.8), married OR 1.9 (1.2-5.2)<br>***Use:*** Education, gravidity, fear of malaria	BV
**Marchant *et al* [25]**	**2010**	**Tanzania**	2027 707p	HH	Assessment of voucher scheme amongst post partum women (n=1320) and currently pregnant women (n=720).	***Use:*** Socioeconomic status 7% ownership amongst poorest (CI 4-13), 48% amongst richest (CI 38-59)	Chi
**Sangare *et al* [70]**	**2012**	**Uganda**	500p	HH	HH surveys of women post partum and pregnant, to examine determinants of ownership and use.	***Use:*** Ownership of >1 ITN RR 1.13 (1-1.3), media advertising bigger influence on use than provision of free ITNs RR 1.5 (1.2-1.8), wealthiest quintile RR 0.77 (0.6- 1), acquisition in pregnancy associated with 100% use.	MV
**Ahmed *et al* [20]**	**2010**	**Uganda**	10234 HH	HH	Possession and use of ITNs in HH with pregnant women and children <5 yo. Assessing equity of an NGO program.	***Use:*** Wealth, urban setting	Chi
**Kolaczinski *et al* [31]**	**2010**	**Uganda**	328p 520 547	HH	Comparing 3 ITN delivery channels to target groups: Routine ANC/ Global Fund for Aids Tuberculosis Malaria funded targeted HH campaign/ USAID funded targeted HH campaign.	***Use:*** Public distribution source of ITN OR 8.4 (4-17.8), large family size>7 OR 1.4 (1.1-1.9)	MV

Legend: p Pregnant women, ANC Antenatal clinic, HH Household, Hosp. Hospital, IPT Intermittent Preventive Treatment, OR Odds Ratio, RR Risk Ratio, CIs 95% Confidence Interval, MV Multivariate, BV Bivariate, UV Univariate, Chi Chi squared analysis, Fisher's Fischer's exact test.
