# Supplementary material for: Ownership and use of insecticide-treated nets during pregnancy in sub-Saharan Africa: a review
Source: Malar J. 2013 Aug 1;12:268. doi: 10.1186/1475-2875-12-268 (PMC3734149; doi:10.1186/1475-2875-12-268)
Supplement: Additional file 4 — Interventions to improve ITN coverage. [file 1475-2875-12-268-S4.pdf]

Additional File 4: Interventions to improve ITN coverage

EVALUATION OF PROGRAMS WITH AN EDUATIONAL COMPONENT

AUTHOR	YEAR	COUNTRY	TYPE	SAMPLE SIZE	STUDY	RESULTS	CONCLUSION
**Muller *et al* [30]**	**2008**	**Burkina Faso**	Cluster RCT	1052 HH<br>(A) 72/107p<br>(B)<br>100/105p	Cluster RCT to evaluate distribution of a specific ITN brand Groups:<br>(A) Free ITNs at ANC + social marketing<br>(B) Social marketing only.	Significant increase in ITN ownership in both groups. (A) 13 to 35% (B) 18 to 23% (significant difference). No difference in ITN use between groups, pre or post intervention.	The free distribution of ITNs to pregnant women through ANCs in addition to ITN social marketing substantially improved ITN household ownership in rural Burkina Faso.
**Pettifor *et al* [26]**	**2009**	**DRC**	Longitudinal	362p<br>328p<br>100p	LLINs + educational leaflet and educational session provided free at first ANC. Women followed up in cohort study to measure ITN use at delivery and post partum.	Ownership 100% after 1st ANC visit. Gains in LLIN use from baseline (25%), to delivery (79%) and post partum (80%). Retention >85%.	Free ITNs are acceptable and result in high usage (? in combination with education).
**Rickard *et al* [27]**	**2011**	**Ghana**	Longitudinal	61 HH<br>13p	Community created and implemented intervention for ITN distribution with education component and monthly follow-up. Change in ITN use measured.	Use increased in pregnant population from 50% to 100% at 12 months.	Universal ITN usage is possible using a community-based approach (although this was small scale).
**Thwing *et al* [32]**	**2008**	**Niger**	Longitudinal	1801 HH<br>254p<br>2450 HH<br>328p	Evaluation of national campaign (+ Red Cross Hang-Up education) for effectiveness, and impact on ITN retention, ownership, and usage (pre and post intervention).	HH ITN Ownership significantly increased from 6% to 65%, as did use in HH with an ITN from 33% to 89%. Retention at 9 months 98%.	The intervention rapidly increased ITN ownership. Retention and usage was high during rainy season.
**Okeibunor *et al* [34]**	**2011**	**Nigeria**	Case control	1280p<br>1380p	Case control in 2 clusters, to determine the effectiveness of a community directed intervention (volunteers to deliver ITNs+ education to pregnant women) versus usual free ITN distribution at ANC.	ITN use during pregnancy significantly higher in test group, compared with control, increasing by 7%, from 27% at baseline. Intervention did not have any effect on ANC attendance.	Community-directed programmes appear to be a cost-effective way to improve malaria prevention. A participatory approach may also strengthen ties between the formal health sector and local communities.
**Bennett *et al* [28]**	**2012**	**Sierra Leone**	Cross section	4260 HH<br>609p	National surveys 6 months post LLIN universal coverage campaign (UCC) with hang up campaign (health fair, education campaign, home visits). LLIN vouchers delivered to HH, pick up point collection.	Ownership of at least 1 ITN increased from 37% (2008) to 88%, and 67% of HH had >1 ITN. Available ITN use 88% in HH with 1 ITN, and 92% in HH with >1 ITN. Education received associated with increased usage rates.	Knowledge of net hanging may be effectively transmitted through the community health workers, and a hanging net is a good predictor of use.
**Gerstl *et al* [33]**	**2010**	**Sierra Leone**	Cross section	137p	Ownership and use of ITNs following MSF distribution of LLINs via mass distribution, delivery and hanging demonstrations.	High levels of ownership (83%) LLIN in HH, and use amongst pregnant women (73%).	MSF achieved good usage with freely distributed LLINs.
**West *et al* [29]**	**2012**	**Tanzania**	Cross section	3246 HH<br>2499 HH<br>228p/ 224p	HH Surveys pre and post UCC. Red Cross and community volunteers provided education and assistance with hanging.	Overall HH Ownership increased from 63% to 91%. Increased usage in pregnant women from 55% to post UCC 63%.	Despite education there remains an ongoing discrepancy between ownership and use of ITNs, though ownership was scaled up.
**Ahmed *et al* [20]**	**2010**	**Uganda**	Cross section	10234 HH	Assessing equity (between peri-urban and rural districts) of a program run by NGO, using community health promoters to distribute subsidized LLINs and health education over 12 months.	No baseline data. Post-intervention data showed poor knowledge about ITNs. HH ownership rate 41- 59% with ITN use at 25-27% amongst pregnant women.	The NGO ITN distribution appeared to be inadequate and inequitable, and their knowledge dissemination was insufficient.

	AUTHOR	YEAR	COUNTRY	TYPE	SAMPLE SIZE	STUDY	RESULTS	CONCLUSION
EVALUATION OF VOUCHER SCHEMES	**Webster *et al* [43]**	**2010**	**Ghana**	Longitudinal	(A) 1232p 1254p (B) 1265p 1226p	Process evaluation of a voucher scheme at ANCs, in 2 villages (A) and (B), pre and post intervention.	Ownership increased in both villages significantly. ANC attendance was high92% (A) and 84% (B). Only 41% (A) and 21% (B) eligible women offered voucher. Cumulative success was 12% (A) and 5% (B)	Voucher scheme was responsible for increase seen in (A) but not in (B), where cumulative success was limited due to gaps in process. Missed opportunity to explore why vouchers not redeemed.
	**Keweku *et al* [23]**	**2007**	**Ghana**	Cross section	25926p	Assessment of a pilot voucher scheme for ITNs.	67% Vouchers redeemed (ownership). Failure to issue vouchers and ITN stock out were major limiting factors, as well as other sources for nets/ competing programs.	More evidence is needed on how specific contextual factors influence the success of voucher schemes, as well as monitoring for voucher issuing and redemption.
	**Marchant *et al* [68]**	**2011**	**Tanzania**	Cross section	363p 883p 915p	Coverage of an antenatal clinic ITN voucher scheme in Tanzania by gestational age from 2005-07 (optimal coverage defined as between 16-40 weeks' gestation).	Overall coverage 84%, optimal coverage 61%. Post intervention use increased from 11 to 23%. Increasing use with increasing gestation observed.	Commencement of malaria interventions is not evenly distributed throughout pregnancy. Including gestational age in pregnancy coverage indicators can enhance our understanding of interventions.
	**Marchant *et al* [25]**	**2010**	**Tanzania**	Cross section	2027 707p	Assessment of voucher scheme via women delivering in the last 12 months (n=1320) and currently pregnant women (n=720).	Inequitable use between SES, with only 23% overall use amongst pregnant women. Biggest drops were between attendance and receiving voucher. Cumulative success 30%.	Gaps in the several steps leading to actual ITN use substantially diminished the overall rate of coverage, particularly amongst the poorest participants.
	**Hanson *et al* [69]**	**2009**	**Tanzania**	Cross section	779p 584p 707p	Progress of household ownership and use of ITNs among target groups after implementation of a national voucher scheme.	Steady increase in ownership (44 to 65%) and use (11 to 23%) over 3 years, including increases in voucher redemption (6 to 24%) in HH with pregnant women.	National Voucher Scheme saw impressive increases in the coverage of ITNs over a two year period. Gaps in coverage remain, however, especially in the poorest groups.
OTHER INTERVENTIONS	**Hightower *et al* [45]**	**2010**	**Kenya**	Cross section	182p	HH use of ITN and ownership in women of reproductive age and pregnant women. Conducted 1 month post mass distribution via measles vaccination campaign.	Ownership in HH increased from 40 to 59%, with available ITN use 57% amongst pregnant women. Retention of nets >95%.	The campaign was successful in reaching the vulnerable populations, but will need to be integrated with other interventions to achieve population coverage targets.
	**Kulkarni *et al* [49]**	**2010**	**Madagasca r**	Cross section	2756 320p	Community based HH survey, evaluating contribution of integrated LLIN distribution campaign to children U5 with other interventions (e.g. vaccination program).	Available ITN use high (89%) post intervention. Campaign was the principle source of nets for pregnant women (72%).	This campaign was found to be equitable and also helped reach coverage targets, particularly amongst vulnerable populations.
	**Skarbinski *et al* [50]**	**2011**	**Malawi**	Cross section	634p	Household surveys in 8 districts to evaluate free health facility based ITN distribution to pregnant women and children <5.	ITN ownership 60% with high use in HH owning nets (90%) by pregnant women.	Targeted campaigns have been successful; however these need to be supplemented with other activities to achieve coverage targets.
	**Mathanga *et al* [51]**	**2009**	**Malawi**	Longitudinal	78p 149p	Surveys pre and post intervention in 2 groups (A&B) with free ITN at U5 vaccination and ANC verses control group (C) with nationally subsidized ITNs.	Ownership significantly increased in test groups, (A) Ownership= 44 to 76% (p=0.01), (B) 57 to 78% (p=0.02), (C) 41 to 48%. Increases were not statistically significant.	Additional studies are required to determine whether this model is effective in other countries, and whether integration with other health services could be synergistic.
	**Kolaczinski *et al* [31]**	**2010**	**Uganda**	Cross section	328p 520 547	Evaluation comparing a one-off campaign directed at HH from fixed points of distribution against routine distribution of free ITNs via ANC with education.	Ownership= >90% (5-7 months post campaign), with high levels of use in HH owning ITNs 74-99%. The use was associated with a public distribution source of the ITN.	Targeted campaigns and routine ANC services can both achieve high LLIN retention and use among the target population. Comparatively higher economic cost of delivery through ANC.

Legend: p Pregnant women, HH Household, UCC Universal coverage campaign, MSF *Médecins Sans Frontières, RCT Randomized Control Trial*
